# Localization of Glucose Transporter 10 to Hair Cells' Cuticular Plate in the Mouse Inner Ear

**DOI:** 10.1155/2018/7817453

**Published:** 2018-06-14

**Authors:** Bei Chen, Yunfeng Wang, Manying Geng, Xi Lin, Wenxue Tang

**Affiliations:** ^1^Department of Otology, The First Affiliated Hospital of Zhengzhou University, Zhengzhou 450000, Henan, China; ^2^Department of Otolaryngology, Eye & ENT Hospital, Fudan University, Shanghai 200031, China; ^3^Departments of Otolaryngology, The Second Affiliated Hospital of Zhengzhou University, Zhengzhou 450000, Henan, China; ^4^Departments of Otolaryngology and Cell Biology, Emory University School of Medicine, Atlanta, GA 30322, USA; ^5^Center for Precision Medicine of Zhengzhou University, Zhengzhou 450052, Henan, China

## Abstract

This study aimed to investigate the localization pattern of glucose transporters (Gluts) in mouse cochlea. Genome-wide gene expression analysis using CodeLink™ bioarrays indicated that Glut1 and Glut10 were highly expressed (~10-fold) in mouse cochlea compared with the other members of glucose transporters (Glut2-6, Glut8, and Glut9). Semiquantitative RT-PCR and western blotting confirmed that Glut10 expression in mouse cochlea was high throughout the embryogenesis and postnatal development. Immunofluorescent staining showed that Glut10 protein was localized in the cuticular plate of the outer and inner cochlear hair cells and in the ampullary crest of the vestibular system. Based on these results, it was supposed that Glut10 may contribute to glucose transport from the endolymph to the hair cells across the cuticular plate.

## 1. Introduction

Inner ear's hair cells are receptor cells that convert mechanical stimuli into electrical signals. These cells are responsible for the transduction of sound stimuli and head movements, enabling the senses of hearing and balance [1]. The stereocilia of the hair cells are the sites of mechanoelectrical transduction. The mechanically gated channels are located near the tip of the F-actin-based stereocilia [2], where tiny vibrations open and close the transduction channels tethered to the cross-linked parallel actin filaments [2].

The cuticular plate (CP) is a stiff actin-based structure that functions as an anchor for the stereocilia. Disrupted actin bundles that form the stereocilia may result in impaired hearing and balance [3]. Nevertheless, the exact CP's roles in hair bundle growth, maintenance, and hearing remain unknown, mainly because the exact proteins present in this unique organelle are unknown [3].

The normal functions of the hair cells require high levels of ATP and consequently glucose for the modulation and maintenance of F-actin and related cytoskeletal proteins [1]. The plasma membrane is impermeable to glucose because glucose is hydrophilic and the lipid bilayer is not. As a result, glucose transport across this membrane requires glucose transporter (GLUT) proteins [4]. The GLUT are membrane transporters that facilitate the transfer of hexoses such as glucose or polyols (e.g., glycerin) across the cellular membrane. The primary physiological substrates and consequently the precise biological function of GLUTs in the inner ear remain unknown. GLUTs generally comprise a 500-amino acid protein backbone and they have a single N-linked oligosaccharide and 12 transmembrane domains. The GLUT family members 1, 2, 3, 4, and 5 have been studied in various tissues and cell types with regard to glucose and/or fructose transport [5].

Recently, a number of previously unknown genes encoding glucose transporter-like proteins have been discovered. The SLC2A gene family encodes 14 members of GLUTs that share a common structure [5]. According to the similarity of their sequence, the members of the GLUT family have been classified as Class I (including GLUT1-4 and GLUT14), Class II (including GLUT5, GLUT7, GLUT9, and GLUT11), and Class III (including GLUT6, GLUT8, GLUT10, GLUT12, and H+-coupled myo-inositol transporter (HMIT)).

The Glut proteins differ in tissue-specific expression. The expression of two members, Glut1 and Glut5, has been reported in the mammal cochlea [6, 7], but other investigators have shown that Glut5 was absent in outer hair cells of the mouse cochlea [8]. Therefore, the present study aimed to investigate the expression pattern of various subtypes of Glut, with a focus on Glut10, and analyze the localization of GluT10 protein in the cochlea and in the ampullary crest.

## 2. Materials and Methods

### 2.1. Animals and Ethics Statement

This study used CD1 male healthy mouse (8 weeks old, purchased from the Jackson Laboratory) without middle ear infection. All aspects of animal care and experiments were conducted according to the National Institutes of Health Guidelines for experimental animals. The study protocol was approved by the Animal Care and Use Committee at Emory University. The animals were sacrificed by cervical dislocation after anesthesia with ketamine (100 mg/kg, i.m.) and xylazine (15 mg/kg, i.m.).

### 2.2. Microarray

The cochlea was dissected and total RNA was isolated using the PicoPure™ RNA Isolation Kit (Catalog# kit0204, Arcturus Bioscience Mt. View, CA, USA). A total of six samples (from six mice) were used.

The RNA quality was measured by an Agilent Bioanalyzer 2100 and RNA 6000 NanoChips (Agilent, Palo Alto, CA). The optimal ratio of 28S:18S band densities was >1.8. Total RNA (100 ng) was used to synthesize the first-strand cDNA.* In vitro* transcription in the presence of biotinylated nucleotides was performed after second-strand cDNA synthesis in order to produce biotin-labeled cRNA. The cRNA concentration was assessed by spectrophotometric analysis prior to hybridization.

cRNA (10 *μ*g) was fragmented, added to CodeLink™ bioarrays (GE Healthcare, Chandler, AZ, USA), and hybridized for 18 h at 37°C under continuous shaking at 300 rpm. After washing the microarrays with 0.75× 0.1 M Tris-HCl (pH 7.6), 0.15 M NaCl, and 0.05% Tween 20 (TNT) at 46°C, Cy5-Streptavidin was added and incubated for 30 min at 25°C to form the streptavidin-dye conjugate. The microarrays were read with a GenePix 4000B scanner (635 nm, 600 V, 100% laser power, 5-*μ*m pixel size, and 0-*μ*m focus position). The images were analyzed by CodeLink Expression Analysis Software (version 4.1, GE Healthcare). The intensity of the signal of each spot was analyzed using GeneSpring 7.3 (Silicon Genetics, Redwood City, CA, USA). Data were normalized according to the median intensity obtained from a single array in order to reduce the interchip variations. The cross-gene error model was active based on the replicates. Data filtering was carried out based on the flags present.

### 2.3. Reverse Transcription-Polymerase Chain Reaction

mRNA expressions of Gluts in cochlea as well as brain, heart, kidney, liver, lung, and pancreas tissues were determined using reverse transcription-polymerase chain reaction (RT-PCR). After dissecting the various tissues, total RNA was isolated the cochlea using the PicoPure™ RNA Isolation Kit (Catalog# kit0204, Arcturus Bioscience Mt. View, CA, USA) and from the other tissues using the Qiagen Rneasy Mini Kit (Qiagen Sciences, Maryland, USA), according to the manufacturers' instructions. Total RNA (0.5 *μ*g) was used for cDNA synthesis using the Applied Biosystems High capacity cDNA Archive kit (Applied Biosystems, Foster City, CA, USA), according to the protocol provided by the manufacturer. The synthesized cDNA (equivalent to 20 ng of total RNA) was used for polymerase chain reaction (PCR) amplification. The Primer Select Vector NTI Advance software program (Invitrogen, Carlsbad, CA, USA) was used to design the primers, which were synthesized by Invitrogen (Carlsbad, CA, USA). The forward and reverse primers are shown in Table 1. The internal control was GAPDH. PCR was conducted using the Gene AMP PCR System (Applied Biosystems, Bedford, MA, USA). PCR conditions were (1) 10-min denaturation step at 95°C (which also activated the Gold variant of Taq polymerase); (2) 35 cycles of amplification that comprised denaturation at 95°C for 30 s, annealing at 55°C for 30 s, and extension at 72°C for 30 s; and (3) final extension for 10 min at 72°C. PCR products were visualized on a 1.5% agarose gel (electrophoresis at 90 mV for 45 min) with ethidium bromide.

### 2.4. Western Blot

The assay was conducted as previously published [9, 10]. Mice were anesthetized and sacrificed using a phosphate-buffered saline (PBS, pH 7.4) intracardial perfusion. Cochleae were dissected in ice-cold PBS, washed twice with cold PBS, and immediately frozen at −80°C. Proteins were extracted using the RIPA buffer (Upstate Biotechnology, Lake Placid, NY, USA), according to the manufacturer's instructions. The protein concentrations were estimated using the bicistronic acid protein assay kit (Pierce, Rockford, IL, USA). The same amounts of proteins (10 *μ*g) were loaded on 4-20% SDS polyacrylamide gels, separated by electrophoresis, and transferred to nitrocellulose membranes (30 V, overnight at 4°C) (Bio-Rad, Richmond, CA, USA). Non-fat dry milk (5% w/v) and Tween 20 (0.1% v/v) in PBS (pH 7.4, 0.12 M) were used to block the membranes for 1 h at 25°C prior to overnight incubation at 4°C with Glut10 primary antibody (1:500 dilution, Alphagenix, GA, USA) in blocking buffer. The membranes were incubated for 1 h at room temperature with the secondary antibody (1:3000 v/v; Bio-Rad, Hercules, CA, USA). The proteins were visualized on a XAR-5 film (Kodak, Rochester, NY, USA) in the presence of the Supersignal West Femto Chemiluminescent Substrate (Pierce, Rockford, IL, USA). The protein abundances were estimated by optical densitometric analysis. The densitometry of each band was determined and analyzed using the Image J software (version 1.61; National Institutes of Health, Bethesda, MD, USA). The background noise was subtracted.

### 2.5. Immunostaining

Mice were anesthetized and sacrificed by intracardial perfusion of 4% v/v paraformaldehyde in PBS (pH 7.4). Inner ears were dissected and were subjected to immunofluorescence staining using either frozen sections or whole mount preparation for 72 h.

In order to conduct immunofluorescence assay in frozen sections, the dissected inner ear tissues were immersed in sucrose (20% v/v in PBS) overnight and embedded overnight in OCT (Sakura Finetek USA Inc., Torrance, CA, USA). The samples were snap frozen in liquid nitrogen and stored at −70°C. Sections (7 *μ*m) were cut on a Cryostat (2800 Frigocut E, Cambridge Instruments GmbH, West Germany). The frozen sections from the ampullary crest of semicircular canals and the Corti organ of mouse cochlea were dissected and permeabilized in PBS-T (0.1% v/v Triton X-100 in PBS) for 30 min. The sections were subsequently blocked for 1 h at room temperature with goat serum (5% in PBS-T). Anti-GluT10 (rabbit IgG, 1:200, Alphagenix, CA, USA) and Anti-Cx26 (rabbit IgG, 1:200, Invitrogen, Carlsbad, CA, USA) were used to label the sections at 4°C overnight. The sections were washed thrice with PBS and incubated for 1 h with secondary antibodies at room temperature. Cy3-conjugated secondary antibodies (anti-rabbit Cy3, 1:400, Jackson ImmunoResearch Lab, West Grove, PA) were used. 4′,6-Diamidino-2-phenylindole (DAPI) was used to stain the nuclei of the hair cells. The sections were mounted with Fluoromount G (Electron Microscopy Sciences, Hatsfield, PA) and examined under a confocal microscope (Zesis LSM, Carl Zeiss USA, Shrewsbury, PA, USA).

A whole mount immunofluorescence assay was performed to detect the expression of Glut10 in the whole mount preparation of cochlear tissues. The cochlear basilar membrane was carefully dissected. This membrane contains the organ of Corti. The specimens were washed thrice in PBS and incubated overnight with anti-Glut10 (rabbit IgG, 1:200, Alphagenix, CA) diluted in goat serum that contained 1% Triton X-100 at 4°C. Following washing thrice with PBS, the secondary antibody was added and incubated for 1 h at room temperature. The following day, the samples were rinsed in PBS for 3 times and immersed in a secondary Alexa Fluor 488 goat anti-rabbit antibody (1:500; Invitrogen, Carlsbad, CA, USA) for 2 h at room temperature. In order to visualize the stereocilia of hair cell, phalloidin conjugated to Alexa568 (1:1000; Sigma, St Louis, MO, USA) was incubated with the samples for 1 h at room temperature for the counterstaining assay. The images were obtained with a laser-scanning confocal microscope (Zeiss LSM, Carl Zeiss).

### 2.6. Statistical Analysis

Data were presented as the means ± SEM and analyzed using Student's t-tests. GraphPad Prism 5 (GraphPad Software Inc., La Jolla, CA, USA) and Origin 7.0 (OriginLab, Northampton, MA, USA) were used for data analysis. Two-sided P-values of less than 0.05 (P<0.05) were considered statistically significant.

## 3. Results

### 3.1. Microarray Analysis Revealed High mRNA Expressions of Glut1 and Glut10 in the Mouse Cochlea

**Figure 1:**
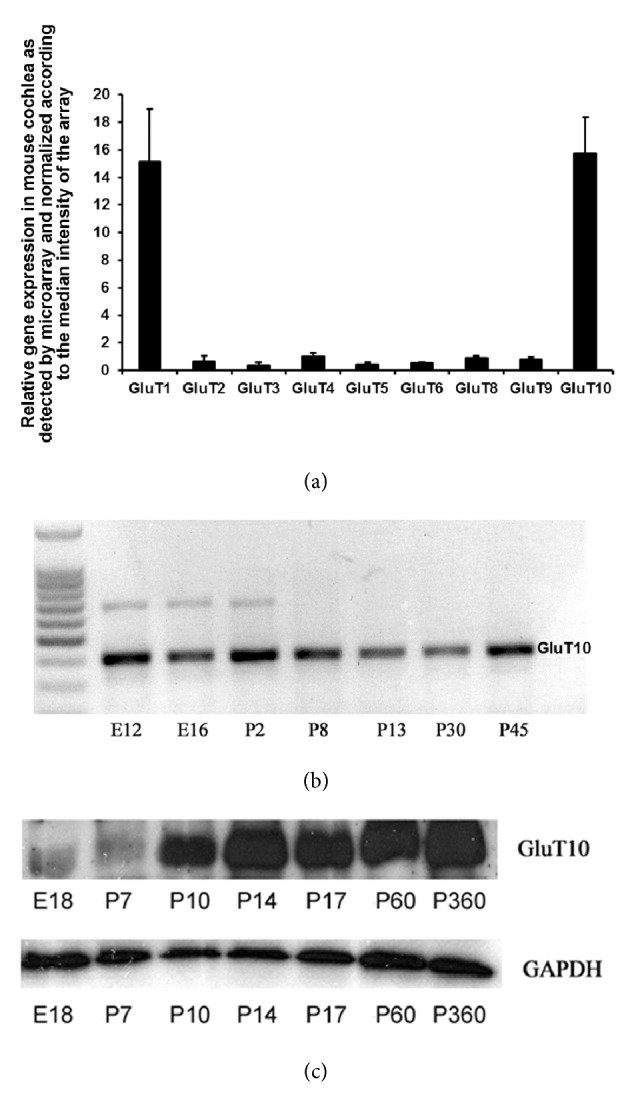
**Expressions of Gluts in the mouse cochlea.** (a) Expression of nine genes of the Glut family in the mouse cochlea (at 8 weeks of age), detected using the CodeLink™ bioarray. The signal intensity for each gene was normalized according to the median intensity obtained from a single array in order to reduce the interchip variation. N=6. SEM: standard error of the mean. (b) The expression of Glut10 in mouse cochlea at different stages of development was measured by semiquantitative RT-PCR. E12/16: embryonic 12/16 days; P2/8/13/30/45: postnatal 2/8/13/30/45 days. (c) The protein amount of Glut10 in mouse cochlea at different stages of development was measured by western blot. The GAPDH was used as the internal control. The representative images are shown. E18: embryonic 18 days; P7/10/14/17/60/360: postnatal 7/10/14/17/60/360 days.

The CodeLink Mouse Whole Genome Bioarray was used to investigate the gene expression profile of the mouse cochlea at 8 weeks of age. A total of 15,156 individual genes were detected with optimal quality flags and were normalized to the median intensity of the array. Nine Glut probes are present on the array (Glut1, Glut2, Glut3, Glut4, Glut5, Glut6, Glut8, Glut9, and Glut10). The results revealed that the expression of Glut1 and Glut10 was high in the mouse cochlea, while the expression of Glut2, Glut3, Glut4, Glut5, Glut6, Glut8, and Glut9 was low (Figure 1(a)).

### 3.2. Temporal and Spatial Analysis of Glut10 Expression

Next, the Glut10 expression in the mouse cochlea was examined over development. The results showed that there were variations in time for Glut10 mRNA expression, but that it was nevertheless highly expressed from E12 to P45 days (Figure 1(b)). At the protein level, Glut10 expression stayed low from E18 to P7 days but increased starting on P10 days and lasting until P360 days (Figure 1(c)).

Besides, the expression pattern of Glut10 was examined in more detail as far as different tissues were concerned. In addition to cochlea, Glut10 mRNA was found in the brain, heart, kidney, liver, lung, and pancreas, and so was the expression pattern of Glut1 at mRNA level (Supplementary Figure 1A). The highest protein expression of Glut10 was found in the pancreas, followed by the lung, liver, heart, cochlea, brain, and kidney (Supplementary Figure 1B).

### 3.3. Location of Glut10 in the Mouse Cochlea and Ampullary Crest

**Figure 2:**
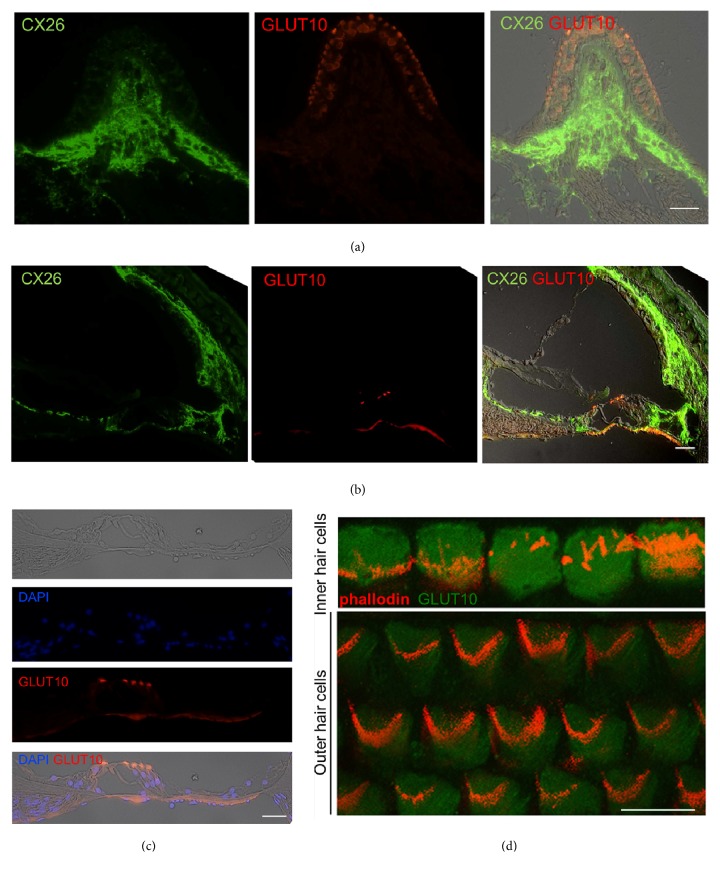
**Location of Glut10 protein in the mouse inner ear.** (a) Coimmunostaining of Glut10 and gap junction protein connexin 26 (CX26) on frozen sections of mouse ampullary crest photographed under a confocal microscope. Scale bar = 20 *μ*m. (b) Coimmunostaining of Glut10 and CX26 on frozen sections of the Corti's organ of mouse cochlea photographed under a confocal microscope. Scale bar = 20 *μ*m. (c) Immunostaining of Glut10 on frozen sections of the Corti's organ of mouse cochlea photographed under a confocal microscope. The nucleus was indicated by DAPI staining. Scale bar = 20 *μ*m. (d) Immunostaining of Glut10 on the whole mount preparation of cochlear tissues photographed under a confocal microscope. The stereocilia of hair cell were marked by staining of actin bundles with phalloidin. Scale bar = 20 *μ*m.

The distribution of Glut10 in the inner ear was examined using coimmunostaining of Glut10 and the gap junction protein connexin 26 (CX26), which is abundantly expressed in the nonsensory cells of the inner ear. The Glut10 protein was predominantly present in sensory epithelial cells of the ampullary crest (Figure 2(a)). No immunoreactivity of Glut10 was detected in the supporting cells and other regions. CX26 staining was mainly present in the region that included nerve fibers below the ampullary crest (Figure 2(a)). A strong immunostaining pattern was observed for CX26 across the cochlear mid-turn, whereas staining of Glut10 was present in the top of the hair cells and in the areas of the cuticular plate, while it was absent from supporting cells, stria vascular, spiral limbus, and spiral ligament (Figure 2(b)). Notably, Glut10 only localized in the cuticular plate both of inner and of outer hair cells, whereas it was absent in the cytoplasm and nuclei of hair cells (Figure 2(c)).

The location of the Glut10 protein in the auditory sensory epithelia was further examined using whole mount immunostaining. It was absent in the stereocilia of hair cell, which was indicated by staining of actin bundles with phalloidin (Figure 2(d)).

## 4. Discussion

In the present study, Glut10 was identified due to its elevated expression levels compared to the other Gluts. Glut10 was localized in the ampullary crest of the vestibular system and in the cuticular plate of the outer and inner hair cells of the cochlea. This is the first study that demonstrates the expression of Glut10 in the mouse cochlea and its localization to the cuticular plate of the outer and inner hair cells.

Previous immunohistochemistry assays of inner ear specimens demonstrated the presence of Glut1 in stria vascularis and vascular endothelium [5, 6]. With regard to Glut10, there are no studies, to the best of our knowledge, that have demonstrated the localization of the protein in the mouse cochlea. A study conducted by Edamatsu et al. [6] demonstrated the mRNA detection of Glut1/3/4/5/8/10/12 and HMIT in the spiral ligament and stria vascularis. Significant differences in gene expression of Glut1/4/5/10 and HMIT between the spiral ligament and stria vascularis were noted, highlighting the differential distribution of the Glut members among various tissue types [6]. The present study corroborates the aforementioned findings and provides novel insight with regard to the immunohistochemical localization of the Glut10 protein in the mouse cochlea. In addition, the expression of Glut5 was not detected by our method, although Glut1 and 10 mRNA levels were clearly detected in the mouse cochlea.

Glut1 localization has been documented in the stria vascularis (SV) and vestibular dark cell areas [10, 11]. In mammals, it is also expressed highly in the blood-brain barrier [12, 13], in atrial tissue (basal side) and small blood vessels such as capillaries [12]. Glut1 is also expressed in the satellite cells encasing spiral ganglion neurons [14]. Absorption of fructose in the intestine requires Glut5 [15]. Glut5 mRNA is also detected in the human brain, kidney, skeletal muscle, and adipocytes. Glut5 immunoreactivity was found as a punctate labeling in the OHCs [7]. Thus it appears that certain differences in the expression pattern of Glut transporters exist among species and within the compartments of the cochlea. Finally, in humans, GLUT10 mRNA has been observed in various organs such as the heart, brain, lung, liver, kidney, pancreas, and placenta. Semiquantitative RT-PCR analysis has further detected GLUT10 mRNA in fetal brain and liver [16, 17].

Glucose transport across the blood-perilymph barrier is a prerequisite for glucose use by the cells [18], but the molecular mechanisms remain to be elucidated. In addition to Gluts, connexins participate in a communication system among cells and play important roles in the exchange of metabolites, electrolytes, and second messengers. The expression of CX26 was demonstrated in many parts of the human inner ear, including the stria vascularis, basement membrane, limbus, and the cochlear spiral prominence [19]. In the cochlea and vestibule, CX26 and CX30 are the main connexins in the mammalian inner ear which are observed in the cells making up the epithelial and connective tissue gap junctions [19].

The exact biological function of Glut10 in the cochlea remains unclear. Glut10 has been shown to be involved in the phenotypic modulation of vascular smooth muscle cells (VSMCs) [20]. The silencing of the Glut10 gene reduces oxidized ascorbic acid transport to the mitochondria, leading to oxidative stress in VSMCs [21]. Mutations in Glut10 are associated with arterial tortuosity syndrome (ATS) [22]. Overexpression or downregulation of Glut10 VSMCs results in vascular remodeling with TGF-*β* signaling activation [23]. Glut10 was initially identified due to its susceptibility loci for noninsulin-dependent diabetes mellitus [24, 25]. Loss of Glut10 in VSMCs leads to lower intracellular glucose concentration [21, 23]. In VSMCs, Lee et al. revealed that Glut10 is also involved in the transport of l-dehydroascorbic acid to the mitochondria, thereby protecting the VSMCs from oxidative stress [21, 23]. We report here Glut10 was localized in the cuticular plate of the hair cells of the cochlea and vestibular system. It could be an indication for a functional role of hair cells in maintaining the supply of glucose in the cuticular plate. In addition, the present study suggests that Glut10 may play a role in hair cell movement, possibly via indirect modulation of the proteins involved in the cuticular plate formation (such as myosin, spectrin, fimbrin, and supervillin) [1]. The biological function of Glut10 in the cochlea still needs to be discovered. Additional studies are necessary to elucidate the exact regulation and effects of Glut10 on hair cell movement. Nevertheless, it has been shown that the remodeling of F-actin in myoblasts is influenced by Glut4 and that PI3K signaling is involved [26]. Aquaporins and Ca^2+^ gradient could also be involved in the regulation of Glut5 [27, 28]. High-energy requirements are necessary for the function of the aforementioned proteins that contribute to the actin polymerization of the cuticular plate [1, 29].

Hair cells in the cochlea play a key role as true mechanoelectrical transducers. The present study provides new data regarding the expression of the transporter Glut10 in the hair cells of mouse cochlea and in the mouse ampullary crest. The data suggest that Glut10 is localized in the cuticular plate and may be important in facilitating its formation and consequently the hair cell movement that in turn affects the normal function of the organ of Corti.

## Figures and Tables

**Table 1:** The forward and reverse primers of Gluts.

Gene	ID	Forward Primer	Reverse Primer	length
Glut1	NM_011400	TGTGTACTGCGGCCTGACTACTG	AACAGCTCCAAGATGGTGACCTTC	399bp
Glut10	NM_130451	TTGCCTCGAACAGGAGCTCC	TAGGAGAGCAGGTGCAGCTG	403bp

## Supplementary Materials

Supplementary Materials Supplementary Figure 1. The expression pattern of Glut10 in mouse different tissues. (A) The expression of Glut1 and Glut10 in cochlea, brain, heart, kidney, liver, lung, and pancreas was measured by semiquantitative RT-PCR. (B) The protein expression of Glut10 in different tissues was measured by western blot. The highest expression was found in the pancreas, followed by the lung, liver, heart, cochlea, brain, and kidney.
