# Supplementary material for: Localization of Glucose Transporter 10 to Hair Cells' Cuticular Plate in the Mouse Inner Ear
Source: Biomed Res Int. 2018 Jun 14;2018:7817453. doi: 10.1155/2018/7817453 (PMC6022331; doi:10.1155/2018/7817453)
Supplement: Supplementary Materials — Supplementary Figure 1. The expression pattern of Glut10 in mouse different tissues. (A) The expression of Glut1 and Glut10 in cochlea, brain, heart, kidney, liver, lung, and pancreas was measured by semiquantitative RT-PCR. (B) The protein expression of Glut10 in different tissues was measured by western blot. The highest expression was found in the pancreas, followed by the lung, liver, heart, cochlea, brain, and kidney. [file 7817453.f1.doc]

**
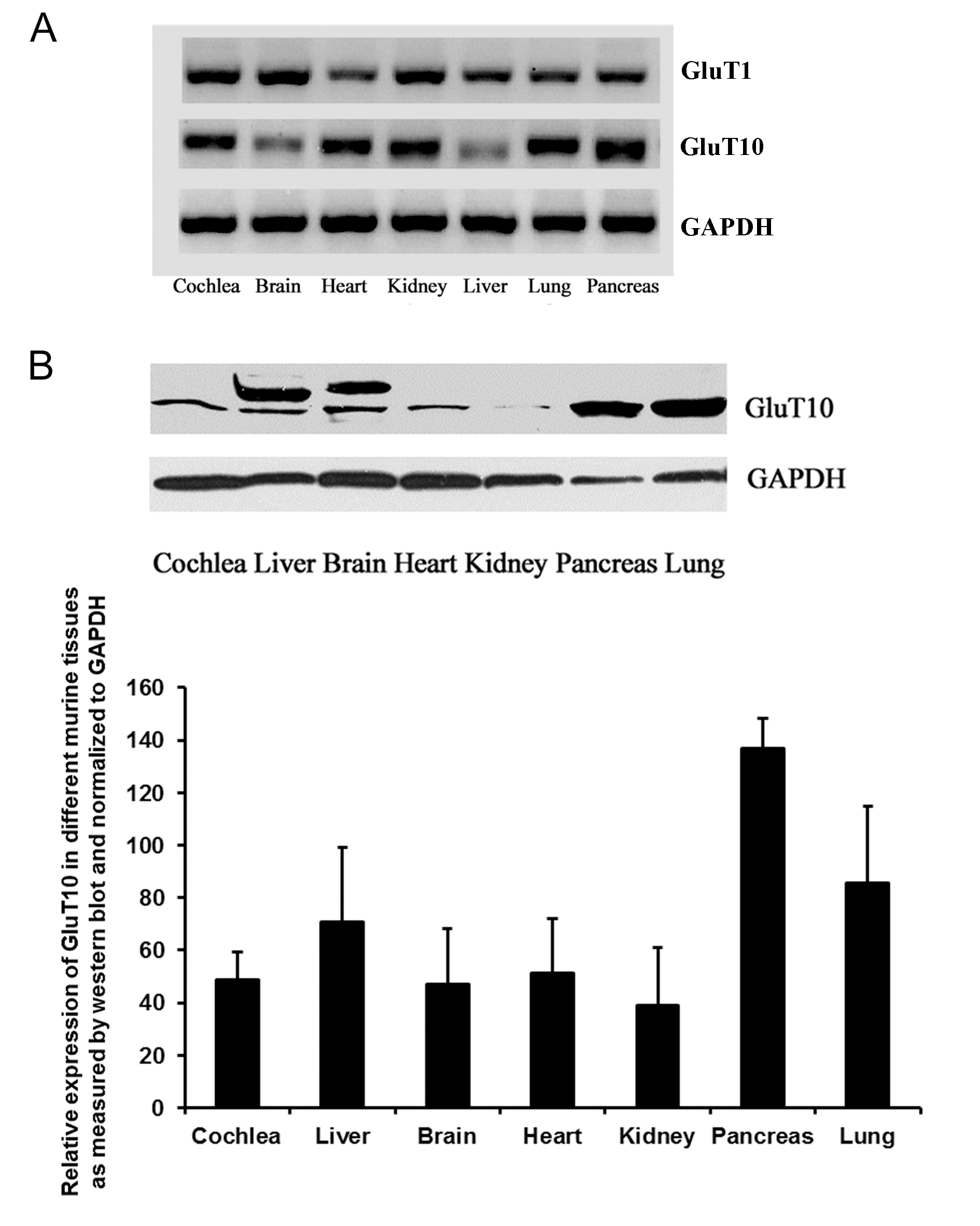
**

**Supplementary Figure 1. Expressions of GluTs in different mouse tissues.**

**(A)** The expressions of GluT1 and Glut10 in different murine tissues (at 8 weeks of age) were measured by semi-quantitative RT-PCR. GAPDH was used as the internal control.

**(B)** The protein amount of GluT 10 in different murine tissues (at the age of 8 weeks old) was measured by western blot. GAPDH was used as the internal control. Shown are the representative images and statistical histograms. N=3. SEM: standard error of the mean.
